# Sedimentary Environment Influences the Effect of an Infaunal Suspension Feeding Bivalve on Estuarine Ecosystem Function

**DOI:** 10.1371/journal.pone.0027065

**Published:** 2011-10-28

**Authors:** Hannah F. E. Jones, Conrad A. Pilditch, Denise A. Bruesewitz, Andrew M. Lohrer

**Affiliations:** 1 Department of Biological Sciences, University of Waikato, Hamilton, New Zealand; 2 National Institute of Water and Atmospheric Research (NIWA), Hamilton, New Zealand; University of Aberdeen, United Kingdom

## Abstract

The suspension feeding bivalve *Austrovenus stutchburyi* is a key species on intertidal sandflats in New Zealand, affecting the appearance and functioning of these systems, but is susceptible to several environmental stressors including sedimentation. Previous studies into the effect of this species on ecosystem function have been restricted in space and time, limiting our ability to infer the effect of habitat change on functioning. We examined the effect of *Austrovenus* on benthic primary production and nutrient dynamics at two sites, one sandy, the other composed of muddy-sand to determine whether sedimentary environment alters this key species' role. At each site we established large (16 m^2^) plots of two types, *Austrovenus* addition and removal. In winter and summer we deployed light and dark benthic chambers to quantify oxygen and nutrient fluxes and measured sediment denitrification enzyme activity to assess denitrification potential. Rates of gross primary production (GPP) and ammonium uptake were significantly increased when *Austrovenus* was added, relative to removed, at the sandy site (GPP, 1.5 times greater in winter and summer; ammonium uptake, 8 times greater in summer; 3-factor analysis of variance (ANOVA), *p*<0.05). Denitrification potential was also elevated in *Austrovenus* addition plots at the sandy site in summer (by 1.6 times, *p*<0.1). In contrast, there was no effect of *Austrovenus* treatment on any of these variables at the muddy-sand site, and overall rates tended to be lower at the muddy-sand site, relative to the sandy site (e.g. GPP was 2.1 to 3.4 times lower in winter and summer, respectively, *p*<0.001). Our results suggest that the positive effects of *Austrovenus* on system productivity and denitrification potential is limited at a muddy-sand site compared to a sandy site, and reveal the importance of considering sedimentary environment when examining the effect of key species on ecosystem function.

## Introduction

Estuaries are highly productive ecosystems that play a major role in biogeochemical cycles, but are subject to multiple stressors that will likely be exacerbated by climate change and expanding human habitation of coastal areas [1], [2], [3]. Although the effects of contaminants, invasive species, coastal alteration and development might be restricted to estuaries near large population centres, enhanced sedimentation rates threaten many estuaries, even when there have been only moderate levels of catchment development [1]. Deposition of large amounts of terrestrial sediments during storm events smother benthic communities, and elevated levels of suspended sediments reduce primary productivity and detrimentally affect suspension feeders (e.g. [4], [5], [6]). More pervasive and perhaps less obvious is the long term degradative change in the form of increasing muddiness that alters estuarine habitats and communities [7], [8].

If habitat change does lead to decreasing biodiversity, then that alone may cause shifts in ecosystem structure and function [9], [10], [11]. However, in many cases it has been shown in estuarine systems that certain key species, rather than biodiversity *per se*, can have a disproportionate effect on indicators of ecosystem functioning such as nutrient cycling and productivity (e.g. [12], [13], [14]). Although the loss of key species likely has important implications, many estuarine species exist across a range of sediment types [8]. Habitat change may not necessarily then cause species loss but might more subtly affect ecosystem function by alteration of a species' functional role. For example an estuarine bioturbating crab (*Austrohelice crassa*) displays functional plasticity, acting as a bioturbator in sandy sediments and as a bioirrigator in muddy cohesive sediments [15]. Thus, the influence of this species on biogeochemical exchange and microbial communities is likely to differ between habitat types [16]. However, most studies to date are restricted temporally and spatially making it difficult to understand the effects of habitat change on a key species' influence on ecosystem function. In this study we examined the effect of a suspension feeding bivalve on ecosystem function at two sites with contrasting sediment properties, in winter and in summer. As sedimentation alters estuarine habitats by increasing sediment mud content we used a site with muddy-sand sediments as a proxy for habitat change, to compare with a site comprising only sandy sediment.

Suspension feeding bivalves can act as key species in estuarine ecosystems by exerting top-down control on phytoplankton populations, affecting rates of nutrient regeneration, contributing to benthic-pelagic coupling, and providing an important food source for higher trophic levels (reviewed by [17]). Furthermore, accumulation of biodeposits and altered redox environments in sediments underlying bivalve beds may enhance sediment denitrification rates, the microbial reduction of NO_3_^−^ to N_2_ gas, which permanently removes fixed nitrogen from an ecosystem; thus, suspension feeding bivalves can also exert a bottom-up control on phytoplankton populations (e.g. [18]). Loss of suspension feeding bivalve populations has resulted in large shifts in ecosystem structure and function. For example, in Chesapeake Bay, USA, loss of eastern oyster (*Crassostrea virginica*) beds has substantially increased the incidence of phytoplankton blooms, sometimes resulting in the occurrence of deep-water hypoxia (e.g. [19], [20]). Conversely, invasion of aquatic systems by non-native suspension feeding bivalves, such as by the Asian clam (*Potamocorbula amurensis*) in San Francisco Bay and the zebra mussel (*Dreissena polymorpha*) in many freshwater systems in the USA, has resulted in reduced phytoplankton biomass (e.g. [21], [22]).

In New Zealand estuaries the dominant suspension feeding bivalve is the native clam *Austrovenus stutchburyi* (hereafter *Austrovenus*), which commonly exists in high density beds covering large areas of intertidal flats; typical bed densities average c. 1000 ind. m^−2^, although peak densities may be 2000–3000 ind. m^−2^ in some areas [23], [24]. *Austrovenus* is an infaunal species that bioturbates surficial sediments through vertical and horizontal movement, but has very short siphons and so lives close to the sediment surface (<5 cm). *Austrovenus* beds are found across a range of sediment types, although very high levels of sedimentation adversely affect abundance [8]. *Austrovenus* has been shown to be a key species influencing sediment stability, solute fluxes and macrofauna community structure as well as enhancing microphytobenthos productivity [12], [25]. However, populations are declining in some areas likely due to chronic sedimentation, pollution and over-harvesting [6], [26], [27].

In this study we manipulated the presence or absence of *Austrovenus in situ* at two estuarine sites, both with nearby high density *Austrovenus* beds, but with contrasting sediment properties. Our aim was to see if the role of this key species in ecosystem functioning was the same at a sandy site (a proxy for a habitat unimpacted by sedimentation) and at a muddy-sand site (a proxy for a habitat affected by a moderate level of sedimentation). In winter and summer, light and dark benthic chambers were used to quantify the effect of *Austrovenus* on O_2_ and nutrient (NH_4_^+^, NO_3_^−^, NO_2_^−^, PO_4_^3−^) fluxes, and to estimate gross primary production and nutrient uptake rates. Additionally, denitrification enzyme activity (DEA) assays were used to quantify the effect of *Austrovenus* on maximum sediment denitrification potential. Previously, high *Austrovenus* densities have been shown to enhance ammonium efflux which supported higher rates of microphytobenthos (MPB) production [25]. Additionally, we expect increased rates of primary production and nutrient cycling in summer compared to winter due to increases in macrofaunal, microbial and photosynthetic activity [8]. Greater retention of bivalve biodeposits was predicted for the more sheltered muddy-sand site. Microbial decomposition of biodeposits may result in enhanced nutrient regeneration and a stimulation of primary production [28]. Alternatively, biodeposit decomposition can elevate denitrification rates through coupled nitrification-denitrification, thus reducing primary production [18]. Our use of large experimental plots (16 m^2^) to reduce confounding edge effects (e.g. [12], [25]) will enhance our understanding of the relative importance of the dynamics of these different habitat types.

## Methods

### Ethics statement

This study complied with all existing legislation governing animal welfare and field-based experiments. Animal ethics approval/permits were not sought as benthic invertebrate fauna manipulated/sampled in this study are exempt from the Animal Welfare Act 1999. After consultation with the Bay of Plenty Regional Council permits were not required for the *in situ* faunal manipulations. The collection of benthic fauna was undertaken with a Ministry of Fisheries Special Permit (386) Client Number 8770024.

### Study site and experimental set up

**Figure 1:**
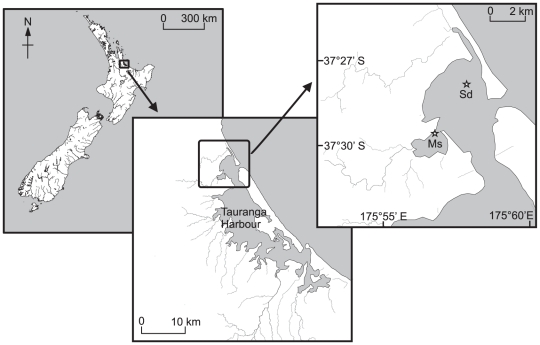
Location of sites (indicated by a star) in Tauranga Harbour, New Zealand. Sd  =  Sandy site, Ms  =  Muddy-sand site.

Tauranga Harbour is a large (200 km^2^) barrier-enclosed estuary on the north-eastern coast of New Zealand. We manipulated the presence or absence of *Austrovenus* at two sites with differing sedimentary characteristics on lower-mid intertidal flats in the harbour (Figure 1). The sandy site (37°27.77′S 175°57.90′E) was located near the northern harbour entrance and was composed of medium sands with no mud content (defined as the silt/clay fraction<63 µm grain size). The muddy-sand site (37°29.20′S 175°56.73′E) was located 3 km up the estuary in the entrance of a small inlet and was composed of fine sands with c. 13% mud content. Mean tidal currents at the sandy site were 13.2 cm s^−1^ (peak flow was 35 cm s^−1^), and at the muddy-sand site were 7.2 cm s^−1^ (peak flow was 18 cm s^−1^), as determined by deployment of a FSI current meter that included a spring and a neap tidal phase. Tides in the harbour are semi-diurnal and the mean immersion period at each site is 8 h. Water temperature in Tauranga Harbour typically fluctuates between 13°C in mid-winter (July/August) and 22°C in mid-summer (January/February) [29].

In June 2009, at both sites, six 4 m×4 m plots separated by 1 m were established in a line parallel with the channel. *Austrovenus* addition and removal treatments were alternated along the transect. The experimental plots were established on areas of sandflat where ambient *Austrovenus* densities were low (c. 300 ind. m^−2^), but were within 20 m of high density *Austrovenus* beds. Preliminary observations indicated that densities in the natural beds were c. 600–1200 ind. m^−2^ at the sandy site, and c. 2000–3000 ind. m^−2^ at the muddy-sand site. We noted however that *Austrovenus* individuals were larger at the sandy site (see results). We intended to raise the density in addition plots so that so that densities were comparable with natural densities for the sites, and so that biomass (and therefore first order excretory and respiration contribution to solute fluxes) was comparable between sites. Therefore, to create the addition treatments (+AS) we collected *Austrovenus* from the nearby natural beds and transplanted them to the plots during the same low tide to raise the density to c. 700 ind. m^−2^ at the sandy site and c. 2000 ind. m^−2^ at the muddy-sand site. Almost all the animals had buried into the sediment by the following day's low tide, and we observed no obvious *Austrovenus* mortality in the days and weeks following the transplants. To create the removal treatments (-AS) we manually removed all *Austrovenus* by finger plowing the sediment, minimising the impact of the manipulation on ambient macrofauna [12], which we repeated the following day to ensure almost total removal. Plastic mesh fences (15 cm in height) were buried 10 cm into the sediment around the perimeter of each plot to prevent the migration of adult *Austrovenus*. The large mesh size (1 cm) and short height (5 cm above sediment) of the fencing was used to minimise effects on water flow [30] and restrictions on the movement of smaller sized macrofauna. The *Austrovenus* manipulation was undertaken 6 weeks prior to the winter (August 2009) and summer (February-March 2010) benthic chamber incubations (see below) to allow the sediment and resident macrofauna to recover from the effects of the manipulation [12].

### In situ chamber incubations

To measure the response of the soft-sediment systems to the *Austrovenus* manipulations, O_2_ and nutrient fluxes were measured in light and dark benthic chambers. One light and one dark chamber was deployed to each of the six plots per site on two consecutive days in both winter and summer. Chambers were placed at least 1 m inside each plot's fence to avoid edge artefacts (e.g. [31], [32]). The four incubations per plot (1 light plus 1 dark on 2 consecutive days) came from four distinct locations so that the same sediments were never resampled. Benthic chamber incubations took place during midday high tides when benthic algal activity was expected to be high.

The incubation chambers (square chambers with domed lids enclosing 0.25 m^2^ sediment and 35 L of mechanically-stirred overlying water) have been described previously [14]. Chamber bases were deployed during the low tide just prior to the incubation, and lids were attached during the incoming tide when water depth was c. 0.5 m. Measurements commenced 2 h before high water and continued for 4 h; *Austrovenus* exhibits a circatidal rhythm whereby feeding is limited to this period [33]. Initially, and once per hour during the incubation, a 60 mL water sample was carefully collected from each chamber using a Luer Lok syringe, without allowing any air bubbles to enter the syringe. O_2_ concentration was measured immediately with a hand held dissolved O_2_ probe (PreSens Fibox 3 PSt3) and the water was then filtered through a Whatman GF/C filter, stored on ice in the dark and frozen that day for later analysis of nutrients (NH_4_^+^, NO_3_^−^, NO_2_^−^, PO_4_^3−^) on a Thermo Scientific Aquakem 200 discrete analyzer. Water column effects on O_2_ and nutrient concentrations were found to be negligible based on incubation of ambient water in light and dark water bottles (1 L) for the same length of time and at the same depth as the chamber incubations. O_2_ and nutrient fluxes were calculated from the slope of the regression between concentration and incubation time, corrected for dilution of chamber water that occurred during each of the five 60 ml samplings. Additionally, HOBO^®^ light meters and TidBit^®^ temperature loggers were fitted to the outside of randomly selected chambers during the experiments.

After chamber deployment 16 surface sediment samples (1 cm depth) were taken from within each chamber footprint using a small syringe core (2.5 cm diameter). Samples were pooled and frozen for later analysis of pigments, grain size, organic matter, nitrogen and organic carbon content. One large core (13 cm diameter, 15 cm depth) was collected for macrofauna analysis, sieved on a 0.5 mm mesh and preserved in 70% isopropyl alcohol with Rose-Bengal stain. A second large core was collected for sediment denitrification and DEA assays (see below) and an additional estimate of *Austrovenus* density (sieved on a 1 mm mesh). For light chamber cores only, the surficial 5 cm of sediment was placed in airtight bags, kept cool and transported to the laboratory that evening for denitrification assays.

### Sediment denitrification assays

Sediment denitrification rates were quantified within 24 h of collection using the chloramphenicol-amended acetylene (C_2_H_2_) inhibition technique [34], [35], [36]. Although this technique results in underestimation of actual denitrification rates due to blocking of nitrification by the C_2_H_2_, it has proven reliable for comparison of denitrification activity among treatments, sites and seasons as well as measuring nutrient limitation of denitrification [e.g. 37,38]. For each sediment sample (5 cm depth core from light chambers) we combined 30 mL of homogenized sediment with 25 mL unfiltered site water in preserve jars modified with *n*-butyl rubber septa in the lids (*n* = 6 per treatment, per site, per season). Chloramphenicol was added to the jars to suppress *de novo* enzyme production and the jars were purged with ultra-pure helium for 10 min to ensure anoxic conditions. Pure C_2_H_2_ was added to the jar headspace to prevent the conversion of N_2_O to N_2_ and gas samples were collected hourly beginning 10 mins after the addition of the C_2_H_2_ for 4 h. To maintain a constant pressure the headspace was replaced with a mixture of helium and C_2_H_2_ after each sample. The gas samples were analysed for N_2_O using a Varian CP 3800 gas chromatograph equipped with a HayeSep D column and electron capture detector. Denitrification rates were calculated from the linear increase in N_2_O concentration over time, normalized to the sediment surface area. To determine whether sediment denitrification was limited by nitrate or carbon we amended additional jars prepared identically to those above with additional nitrate (as potassium nitrate 10 mg N L^−1^), carbon (as glucose 12 mg C L^−1^) or both nitrate (10 mg N L^−1^) and carbon (12 mg C L^−1^). The DEA measurements were determined from the rates measured in the samples amended with nitrate and carbon (+N+C). DEA provides a measure of maximum denitrification potential by providing optimized conditions in anoxic, +N+C-amended slurries, valuable for making across-site comparisons [39], [40].

### Laboratory analyses

Sediment chlorophyll *a* (chl *a*) and phaeopigment content were determined by extraction in 90% acetone and measurement of fluorescence before and after acidification on a Turner Designs 10-AU fluorometer [41]. Organic matter content (OM) was determined from dried (60°C for 24 h) and ashed (550°C for 4 h) sediment samples. Sediment grain size was measured on a Malvern Mastersizer-S after preparing the samples with 10% hydrogen peroxide to remove OM, removal of the >1 mm fraction, and addition of calgon to disperse the particles [42]. Organic carbon (OC) and total nitrogen (N) was measured on a LECO CHN analyser after removal of carbonate carbon from the samples by acidification with 1M hydrochloric acid [43]. Macrofauna samples were sorted into six broad taxonomic groups; *Austrovenus*, other bivalves, mudflat anemones (*Anthopleura aureoradiata)*, annelids, crustaceans and gastropods counted and weighed (blotted wet weight). The weight of the bivalves included their shells. *Austrovenus* density and biomass in each chamber was estimated from the mean of the two large sediment cores.

### Data analysis

To eliminate pseudo-replication, one representative value for each chamber type per plot was obtained prior to statistical analysis by averaging the data from the two light and two dark chambers deployed per plot. Sediment O_2_ and nutrient fluxes in the light and dark chambers were analysed separately. We defined the rate of net primary production (NPP) and sediment oxygen consumption (SOC) as the O_2_ flux in light and dark chambers respectively and estimated gross primary production (GPP) from NPP-SOC. GPP was standardised by the sediment chl *a* content to account for variations in microphytobenthos biomass. We estimated nutrient uptake rates (the difference between dark chamber flux and light chamber flux) to quantify usage by microbes and microphytes living in surficial sediments.

The response variables (NPP, SOC, GPP, nutrient fluxes and uptake, and sediment DEA) were analysed using 3-factor analysis of variance (ANOVA), with treatment (+AS, -AS), site (sandy, muddy-sand), and season (winter, summer) all considered as fixed factors. Any non-significant interaction terms of the highest order were removed and the analysis repeated. When the overall ANOVA was significant at α = 0.05, pairwise comparisons were performed using Tukey post-hoc tests. For sediment denitrification, 2-factor ANOVA by presence or absence of nitrate (N) or carbon (C) was used to identify the limiting nutrient. Single nutrient limitation by N or C is identified with a significant result for that treatment, and co-limitation is identified by a significant interaction term [44]. One-factor ANOVA were used to compare sediment properties, *Austrovenus* density and biomass between sites, seasons and treatments separately. In all tests, normality and homogeneity of variances were evaluated with Kolmogorov-Smironov tests and by plotting of residual versus predicted values. Variables were log or square root transformed where required. All statistical analyses were performed using Statistica (Version 8, Statsoft Inc., 2008).

## Results

### Macrofauna abundance and biomass

**Figure 2:**
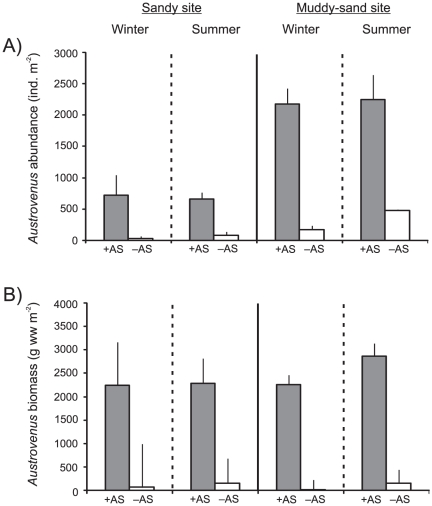
*Austrovenus stutchburyi* abundance and biomass. Mean (+ 1 SD; n = 3) *Austrovenus* abundance (A) and biomass (B) in *Austrovenus* addition (+AS; grey fill) and removal (–AS; no fill) plots as a function of site and season.

*Austrovenus* density in +AS plots ranged from c. 500 to 1000 ind. m^−2^ at the sandy site and from c. 1800 to 2500 ind. m^−2^ at the muddy-sand site (Figure 2). Small-scale spatial heterogeneity in *Austrovenus* density is characteristic of natural *Austrovenus* beds, as the adults tend to be aggregated rather than randomly or uniformly distributed (e.g. [24]). However, we expected the large size of our experimental plots (16 m^2^) to affect the sediment biogeochemical environment at a scale larger than the chamber footprints (0.25m^2^). Although we did not achieve total removal in -AS plots, *Austrovenus* density and biomass were at least an order of magnitude less than in the +AS plots.

Regardless of site or season, *Austrovenus* density and biomass were significantly greater in +AS compared to -AS plots (1-factor ANOVA, *p*<0.001). Densities in +AS plots were equivalent to planned densities, i.e. mean *Austrovenus* density in +AS plots was significantly lower at the sandy site (700 ind. m^−2^) compared to the muddy-sand site (2000 ind. m^−2^, *p*<0.001). Mean *Austrovenus* shell length (± SD) was significantly greater at the sandy site, 23.3 (±1.0) mm, compared to the muddy-sand site, 17.7 (±1.1) mm, (*p*<0.001). Thus, as expected, mean biomass in +AS plots (c. 2300 g ww m^−2^) was not significantly different between the two sites (*p*>0.05). There was no significant seasonal difference in *Austrovenus* density, size or biomass at either site (*p*>0.05).

**Figure 3:**
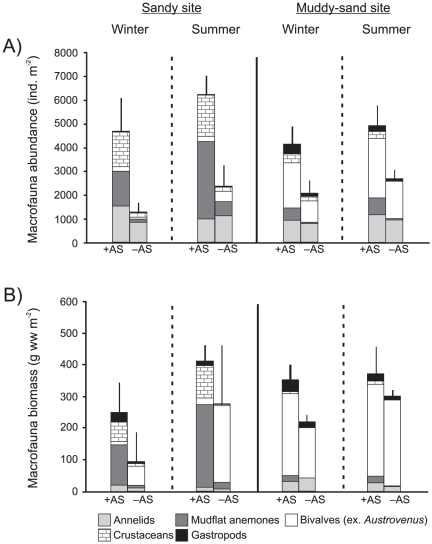
Macrofauna (excluding *Austrovenus stutchburyi*) abundance and biomass. Mean (+ 1 SD; n = 3) macrofauna abundance (A) and biomass (B) in *Austrovenus* addition (+AS) and removal (–AS) plots as a function of site and season.

Abundance of other macrofaunal groups was dominated by annelids, *Anthopleura aureoradiata* (mudflat anemones, attached to the *Austrovenus* shells) and crustaceans (mostly barnacles, also attached to the *Austrovenus* shells) at the sandy site; annelids and other bivalves dominated at the muddy-sand site (Figure 3A). *Austrovenus* comprised c. 90% of the mean total macrofaunal biomass in the +AS plots. Other than *Austrovenus* the biggest contributors to macrofaunal biomass were *Anthopleura* in +AS plots at the sandy site, other bivalves in -AS plots at the sandy site, and other bivalves in both +AS and -AS plots at the muddy-sand site (Figure 3B).

### Environmental variables

There were large differences in water temperature and photosynthetically active radiation (PAR) between winter and summer, with only small differences between sites within each of the seasons. Conversely, there were large differences in sediment properties between sites, but not between winter and summer (Table 1). Water temperature was greater in summer (c. 22°C) than in winter (c. 14°C). Levels of PAR were also much greater in summer (c. 1370 µmol photons m^−2^ s^−1^) than in winter (c. 80 µmol photons m^−2^ s^−1^). Regardless of season or *Austrovenus* treatment, median grain size was significantly lower at the muddy-sand site (c. 220 µm), compared to the sandy site (c. 420 µm, 1-factor ANOVA, *p*<0.001). Mud, OM, OC, N, chl *a* and phaeopigment content were all significantly greater at the muddy-sand site (*p*<0.001). We did not detect a significant effect (α = 0.05) of *Austrovenus* treatment on any sediment properties at the sandy site. However, at the muddy-sand site, in both winter and summer, grain size was greater (*p*<0.05) and mud content was lower (*p*<0.05) in +AS than -AS plots. Also at the muddy-sand site, OM content was lower in +AS plots than in -AS plots, although the effect was only marginally significant (*p* = 0.088 in winter, *p* = 0.075 in summer).

**Table 1:** Environmental variables as a function of site, season and treatment.

Environmental variable	Treatment	Sandy Site		Muddy-sand site	
		Winter	Summer	Winter	Summer
Median grain size (µm)	+AS	447 (38)	393 (20)	222 (8)	262 (14)
	–AS	463 (50)	389 (62)	195 (15)	221 (14)
Silt/clay (%)	+AS	0.0 (0.0)	0.0 (0.0)	10.8 (0.4)	9.1 (1.2)
	–AS	0.0 (0.0)	0.6 (1.0)	17.0 (2.0)	13.6 (2.1)
Organic matter (%)	+AS	1.1 (0.1)	1.0 (0.1)	3.7 (0.3)	3.2 (0.1)
	–AS	1.2 (0.2)	1.1 (0.3)	4.2 (0.3)	3.4 (0.1)
Chlorophyll *a* (µg g dw^−1^)	+AS	8.4 (0.5)	8.5 (1.9)	23.7 (1.3)	17.7 (0.7)
	–AS	8.6 (0.6)	8.2 (3.6)	22.0 (1.6)	14.5 (1.8)
Phaeopigment (µg g dw^−1^)	+AS	2.5 (0.2)	1.4 (0.5)	14.3 (0.4)	7.3 (0.4)
	–AS	2.5 (0.2)	1.6 (1.0)	15.9 (1.9)	6.0 (0.7)
Organic carbon (%)	+AS	0.15 (0.00)	0.17 (0.01)	0.37 (0.02)	0.31(0.01)
	–AS	0.16 (0.01)	0.18 (0.02)	0.45 (0.07)	0.34(0.04)
Nitrogen (%)	+AS	0.08 (0.00)	0.09 (0.00)	0.14 (0.01)	0.12(0.01)
	–AS	0.08 (0.01)	0.09 (0.01)	0.14 (0.01)	0.12(0.01)
OC:N	+AS	2.0 (0.1)	2.0 (0.2)	2.6 (0.2)	2.7 (0.1)
	–AS	2.1 (0.2)	2.1 (0.0)	3.2 (0.4)	2.8 (0.2)
Water temperature (°C)		13.9 (13.5 – 14.2)	21.4 (21.0 – 21.6)	14.7 (14.2 – 15.0)	22.6 (22.0 – 23.1)
PAR (µmol photons m^−2^ s^−1^)		82 (58 – 105)	1330 (560 – 2100)	81 (68 – 93)	1410 (1330 – 1490)

+AS  =  *Austrovenus* addition, –AS  =  *Austrovenus* removal, PAR  =  photosynthetically active radiation, OC:N  =  organic carbon to nitrogen ratio. For water temperature and PAR data represent mean and range in parentheses measured during chamber incubations. For sediment properties data represent mean (n = 3) with SD in parentheses.

### O_2_ fluxes and GPP

**Figure 4:**
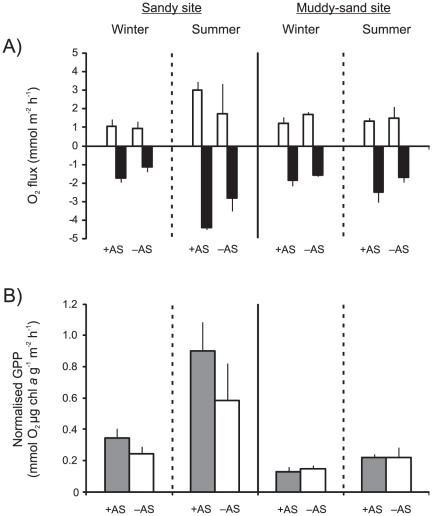
O_2_ fluxes and gross primary production (GPP). (A) Mean (+ 1 SD; n = 3) O_2_ fluxes in light (no fill) and dark (black fill) chambers in *Austrovenus* addition (+AS) and removal (–AS) plots, as a function of site and season. Positive values represent an efflux out of the sediment, and negative values represent an influx into the sediment. (B) Mean (+ 1 SD; n = 3) normalised GPP (light minus dark chamber O_2_ flux) in +AS (grey fill) and –AS (no fill) plots, as a function of site and season.

In dark chambers there was always an influx of O_2_ into the sediments, indicating sediment oxygen consumption (SOC), however, in light chambers there was always an efflux of O_2_ from the sediments which indicated that net primary production (NPP) was greater than zero (Figure 4A). There was a significant treatment effect on SOC which was 1.5× higher in +AS plots compared to -AS plots (3-factor ANOVA, *p*<0.001, Table 2). Post-hoc analysis of the site*season interaction (*p*<0.001) revealed that SOC was significantly greater (by 2.5×) in summer than in winter at the sandy site but that there was no significant difference between seasons at the muddy-sand site. Comparisons between sites within seasons demonstrated that SOC was significantly higher (by 1.7×) at the sandy than at the muddy-sand site in summer only (in winter there was no significant difference). For light chamber O_2_ fluxes, there was a marginally significant site*treatment interaction (*p* = 0.086). Closer examination suggested that NPP tended to be greater in +AS plots (compared to -AS plots) at the sandy site in summer. There was no indication of treatment effects on NPP in winter at the sandy site or at the muddy-sand site in either season. The site*season interaction was significant (*p*<0.05) with NPP greater (by 2.4×) in summer than in winter at the sandy site. There was no significant seasonal effect on NPP at the muddy-sand site and no significant difference between the sites in either season.

**Table 2:** 3-factor ANOVA (analysis of variance) results for sediment oxygen consumption (SOC; dark chamber O_2_ flux), net primary production (NPP; light chamber O_2_ flux) and gross primary production normalised by sediment chl *a* content (GPP/chl *a*).

Variable	Source	d.f.	MS	F	*p*	Significant Tukey post-hoc test		
						Site	Season	Treatment
SQRT SOC	Site	1	0.170	12.9	**0.002**			
	Season	1	1.02	78.2	**< 0.001**			
	Treatment	1	0.422	32.2	**< 0.001**			+AS>–AS
	Site*Season	1	0.482	36.8	**< 0.001**	Su: Sd>Ms	Sd: Wi<Su	
	Site* Treatment	1	0.0287	2.19	0.157			
	Season* Treatment	1	0.0455	3.47	0.080			
	Error	17	0.0131					
NPP	Site	1	0.364	0.800	0.383			
	Season	1	2.65	5.83	**0.027**			
	Treatment	1	0.205	0.451	0.511			
	Site*Season	1	2.96	6.51	**0.021**		Sd: Wi<Su	
	Site* Treatment	1	1.51	3.32	0.086			
	Season* Treatment	1	0.917	2.02	0.174			
	Error	17	0.455					
SQRT GPP/chl *a*	Site	1	0.457	86.3	**< 0.001**			
	Season	1	0.246	46.4	**< 0.001**			
	Treatment	1	0.024	4.61	**0.047**			
	Site*Season	1	0.071	13.5	**0.002**	Su & Wi: Sd>Ms	Sd: Wi<Su	
	Site* Treatment	1	0.035	6.60	**0.020**	+AS & –AS: Sd>Ms		Sd: +AS>–AS
	Season* Treatment	1	0.006	1.17	0.295			
	Error	17	0.005					

Factors are site (Sd  =  Sandy, Ms  =  Muddy-sand), season (Wi  =  Winter, Su  =  Summer) and treatment (+AS  =  *Austrovenus* addition, –AS  =  *Austrovenus* removal). Values in bold are significant at *p*<0.05. Tukey post-hoc tests for significant differences between site, season and treatment are shown at α = 0.05. SOC and GPP/chl *a* were square root (SQRT) transformed prior to analysis.

Mean GPP ranged from 2.1 to 7.4 mmol O_2_ m^−2^ h^−1^ at the sandy site, but the range was much smaller at the muddy-sand site (3.1 to 3.8 mmol O_2_ m^−2^ h^−1^). When normalised by sediment chl *a* content (a proxy for primary producer biomass), GPP at the sandy site was consistently greater (0.24 to 0.90 mmol O_2_ µg chl *a* g^−1^ m^−2^ h^−1^) than at the muddy-sand site (0.13 to 0.22 mmol O_2_ µg chl *a* g^−1^ m^−2^ h^−1^, Figure 4B). There were significant site*season and site*treatment interaction effects on normalised GPP (3-factor ANOVA, *p*<0.05, Table 2). Post-hoc analysis showed that normalised GPP was higher in +AS plots compared to –AS plots at the sandy site, (by 1.4× in winter and by 1.5× in summer), but there was no significant difference between the treatments at the muddy-sand site in either season. Between sites within season comparisons demonstrated that normalised GPP was greater at the sandy site in both winter (by 2.1×) and summer (by 3.4×). Comparison between seasons within sites demonstrated that normalised GPP was greater at the sandy site in summer compared to winter (by 2.6×), but there was no significant difference between winter and summer at the muddy-sand site.

### Nutrient fluxes and uptake

**Figure 5:**
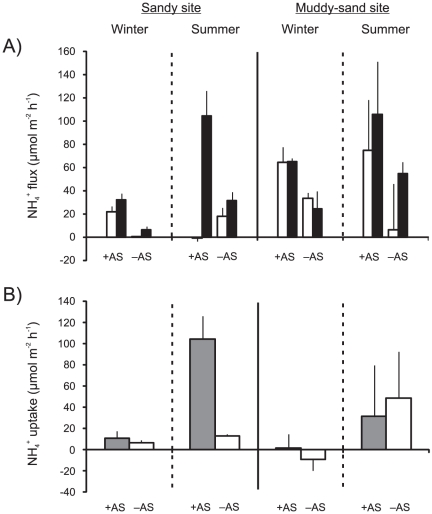
NH_4_^+^ fluxes and uptake. (A) Mean (+ 1 SD; n = 3) NH_4_^+^ fluxes in light (no fill) and dark (black fill) chambers in *Austrovenus* addition (+AS) and removal (–AS) plots, as a function of site and season. Positive values represent an efflux out of the sediment, and negative values represent an influx into the sediment. (B) Mean (+ 1 SD; n = 3) NH_4_^+^ uptake (dark minus light chamber NH_4_^+^ flux) in +AS (grey fill) and –AS (no fill) plots, as a function of site and season.

In dark and light chambers there was nearly always a net efflux of ammonium (NH_4_^+^) from the sediment, the only exception being some light chambers in the +AS plots at the sandy site in summer, when there was a small influx (Figure 5A). There was a significant treatment effect on dark chamber NH_4_^+^ flux which was 2.6× greater in +AS plots compared with –AS plots (3-factor ANOVA, *p*<0.001, Table 3). Post-hoc analysis of the site*season interaction (*p*<0.05) showed that dark chamber NH_4_^+^ flux was greater in summer than in winter at both sites (by 1.8× at the muddy-sand site, and by 3.6× at the sandy site). Comparisons between sites within seasons demonstrated that dark chamber NH_4_^+^ flux was greater (by 2.3×) at the muddy-sand site than at the sandy site in winter only (in summer there was no significant difference). The effect of *Austrovenus* treatment on light chamber NH_4_^+^ flux was not consistent across sites and seasons (3-factor ANOVA, *p*<0.05, Table 3). Post-hoc analysis of the site*season*treatment interaction revealed that light chamber NH_4_^+^ flux was significantly greater (by 11.8×) in +AS compared to -AS plots at the muddy-sand site in summer, but there was no significant difference between treatments in winter or at the sandy site in either season. Comparison between sites within seasons and treatments revealed that in +AS plots in summer light chamber NH_4_^+^ flux was significantly greater at the muddy-sand site; at the sandy site NH_4_^+^ flux was negative (−0.52 µmol m^−2^ h^−1^) indicating a small influx into the sediment, but at the muddy-sand site NH_4_^+^ flux was positive (74.6 µmol m^−2^ h^−1^) indicating a large efflux out of the sediment. In contrast, in +AS plots in winter and in -AS plots in both seasons, there was no significant difference between the sites.

**Table 3:** 3-factor ANOVA (analysis of variance) results for dark and light chamber NH_4_^+^ flux and NH_4_^+^ uptake.

Variable	Source	d.f.	MS	F	*p*	Significant Tukey post-hoc test		
						Site	Season	Treatment
Log_10_ Dark NH_4_^+^	Site	1	0.464	16.4	**< 0.001**			
	Season	1	1.24	44.0	**< 0.001**			
	Treatment	1	1.52	53.7	**< 0.001**			+AS>–AS
	Site*Season	1	0.161	5.70	**0.029**	Wi: Sd<Ms	Sd & Ms: Wi<Su	
	Site* Treatment	1	0.105	3.72	0.071			
	Season* Treatment	1	0.078	2.75	0.116			
	Error	17	0.028					
Light NH_4_^+^	Site	1	7190	15.3	**0.001**			
	Season	1	172	0.366	0.554			
	Treatment	1	3850	8.19	**0.011**			
	Site*Season	1	58.7	0.125	0.728			
	Site* Treatment	1	3470	7.39	**0.015**			
	Season* Treatment	1	2.55	0.005	0.942			
	Site*Season*Treatment	1	2290	4.88	**0.042**	+AS Su: Sd<Ms		Ms Su: +AS>–AS
	Error	16	470					
NH_4_^+^ uptake	Site	1	1430	2.28	0.151			
	Season	1	13400	21.4	**< 0.001**			
	Treatment	1	2950	4.69	**0.046**			
	Site*Season	1	66.0	0.110	0.749			
	Site* Treatment	1	3980	6.32	**0.023**			
	Season* Treatment	1	1320	2.09	0.168			
	Site*Season* Treatment	1	4990	7.92	**0.012**	+AS Su: Sd>Ms	+AS Sd: Wi<Su	Sd Su: +AS>–AS
	Error	16	629					

Factors are site (Sd  =  Sandy, Ms  =  Muddy-sand), season (Wi  =  Winter, Su  =  Summer) and treatment (+AS  =  *Austrovenus* addition, –AS  =  *Austrovenus* removal). Values in bold are significant at *p*<0.05. Tukey post-hoc tests for significant differences between site, season and treatment are shown at α = 0.05.

Dark NH_4_^+^ flux was log_10_ transformed prior to analysis.

NH_4_^+^ uptake exhibited a far greater range at the sandy (6 to 105 µmol NH_4_^+^ m^−2^ h^−1^) compared to the muddy-sand site (−9 to 49 µmol NH_4_^+^ m^−2^ h^−1^; Figure 5B). The effect of *Austrovenus* treatment on NH_4_^+^ uptake was inconsistent across sites and seasons (3-factor ANOVA, *p*<0.05, Table 3). Post-hoc analysis of the site*season*treatment interaction demonstrated that NH_4_^+^ uptake was significantly increased (by 8×) in +AS compared to -AS plots at the sandy site in summer, but there was no significant difference between treatments in winter. At the muddy-sand site there was no treatment effect in either season. Comparison between seasons within treatments and sites revealed that in +AS plots at the sandy site NH_4_^+^ uptake was significantly greater (by 10×) in summer compared to winter, but there was no significant difference between the seasons in -AS plots. At the muddy-sand site there was no significant difference between the seasons in +AS or -AS plots. Comparison between sites within treatments and seasons revealed that NH_4_^+^ uptake was significantly greater (by 3.4×) at the sandy site in +AS plots in summer, but there was no significant difference between the sites in +AS plots in winter. There was also no significant difference between the sites in -AS plots in either season.

**Figure 6:**
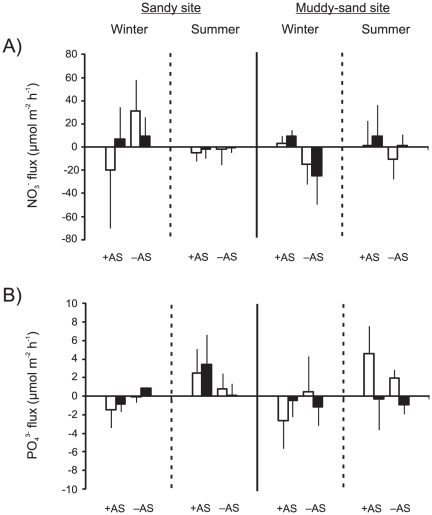
NO_3_^−^ and PO_4_^3^^−^ fluxes. Mean (+ 1 SD; n = 3) NO_3_^−^ (A) and PO_4_^3−^ (B) fluxes in light (no fill) and dark (black fill) chambers in *Austrovenus* addition (+AS) and removal (–AS) plots, as a function of site and season. Positive values represent an efflux out of the sediment, and negative values represent an influx into the sediment.

NH_4_^+^ is the form of dissolved inorganic nitrogen (DIN) most readily taken up by microphytobenthos (MPB) and, as is typically the case in New Zealand estuaries, comprised the majority (c. 80%) of DIN in our samples [12], [25], [45]. We measured high variation in NO_3_^−^ and PO_4_^3−^ fluxes (Figure 6). Additionally, chamber nutrient concentrations were often near instrument detection limits, particularly for NO_2_^−^ (0.005 µmol L^−1^), and this led to uncertainty in flux estimates (mean r^2^<0.3). There were no obvious treatment effects and no further analyses were conducted for NO_3_^−^, NO_2_^−^ or PO_4_^3−^ fluxes.

### Sediment denitrification rates

Non-amended denitrification rates (0 to 30 µmol N m^−2^ h^−1^) were lower than the sediment denitrification potential (38 to 164 µmol N m^−2^ h^−1^), which was determined from samples amended with nitrate and carbon (DEA). Two-way ANOVA revealed a significant effect of nitrate addition at both sites in winter and in summer, indicating that denitrification was always N limited, regardless of site or season (Table 4).

**Table 4:** 2-factor ANOVA (analysis of variance) results determining whether nitrogen, carbon or both nutrients are limiting dentrification rates.

Site/Season	Source	d.f.	MS	F	*p*	Significant Tukey post-hoc test	
						Nitrogen	Carbon
Sandy site in winter	Nitrogen	1	189	193	**< 0.001**		
	Carbon	1	8.05	8.23	**0.009**		
	Nitrogen*Carbon	1	6.74	6.89	**0.016**	+C & –C: +N>–N	–N: –C>+C
	Error	20	0.980				
Sandy site in summer	Nitrogen	1	92000	97.8	**< 0.001**	+N>–N	
	Carbon	1	548	0.582	0.454		
	Nitrogen*Carbon	1	548	0.582	0.454		
	Error	20	942				
Muddy-sand site in winter	Nitrogen	1	256	447	**< 0.001**	+N>–N	
	Carbon	1	5.61	9.77	**0.005**		–C>+C
	Nitrogen*Carbon	1	0.226	0.394	0.537		
	Error	20	0.574				
							
Muddy-sand site in summer	Nitrogen	1	8640	291	**< 0.001**	+N>–N	
	Carbon	1	56.5	1.90	0.183		
	Nitrogen*Carbon	1	56.5	1.90	0.183		
	Error	20	29.7				

Factors are nitrogen (N) and carbon (C). Values in bold are significant at *p*<0.05. Tukey post-hoc tests for significant differences between presence/absence (+/–) of nitrogen and carbon are shown at α = 0.05.

**Figure 7:**
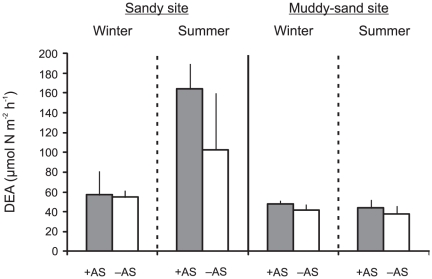
Sediment DEA (denitrification enzyme activity; i.e. denitrification potential). Mean (+ 1 SD; n = 3) DEA in *Austrovenus* addition (+AS; grey fill) and removal (–AS; no fill) plots as a function of site and season.

As with GPP and NH_4_^+^ uptake, the range in sediment denitrification potential was greater at the sandy site (55 to 164 µmol N m^−2^ h^−1^) compared to the muddy-sand site (38 to 48 µmol N m^−2^ h^−1^; Figure 7). Denitrification potential did trend towards an increase in +AS compared to -AS plots at the sandy site, especially in summer, although the treatment effect was only marginally significant (3-factor ANOVA, *p* = 0.078, Table 5). There was a significant site*season interaction (*p*<0.001) and post-hoc analysis demonstrated that denitrification potential was significantly greater (by 2.4×) in summer compared to winter at the sandy site, but there was no significant seasonal effect at the muddy-sand site. Also, denitrification potential was significantly greater (by 3×) at the sandy site than at the muddy-sand site in summer, but there was no significant difference between the sites in winter.

**Table 5:** 3-factor ANOVA (analysis of variance) results for log_10_ transformed DEA (denitrification enzyme activity; i.e. sediment denitrification potential).

Variable	Source	d.f.	MS	F	*p*	Significant Tukey post-hoc test		
						Site	Season	Treatment
Log_10_ DEA	Site	1	0.486	34.8	**< 0.001**			
	Season	1	0.145	10.4	**0.005**			
	Treatment	1	0.049	3.52	0.078			
	Site*Season	1	0.242	17.3	**< 0.001**	Su: Sd>Ms	Sd: Wi<Su	
	Site* Treatment	1	0.004	0.310	0.585			
	Season* Treatment	1	0.023	1.62	0.220			
	Error	17	0.014					

Factors are site (Sd  =  Sandy, Ms  =  Muddy-sand), season (Wi  =  Winter, Su  =  Summer) and treatment (+AS  =  *Austrovenus* addition, –AS  =  *Austrovenus* removal). Values in bold are significant at *p*<0.05. Tukey post-hoc tests for significant differences between site, season and treatment are shown at α = 0.05.

## Discussion

At the sandy site, there were significant increases in many response variables (i.e. SOC, NPP, GPP, NH_4_^+^ uptake and denitrification potential) in summer, compared to winter. In contrast, for the same variables at the muddy-sand site, there was no significant difference between winter and summer measurements. Similarly, the effect of *Austrovenus* treatment on response variables was inconsistent between sites and seasons. Although both SOC and dark chamber NH_4_^+^ fluxes increased significantly in +AS plots regardless of site and season, GPP (and NPP to a lesser extent) were increased in +AS plots only at the sandy site. An increase in GPP indicates increased MPB productivity as water column primary production was negligible. Our results suggest that increased availability of NH_4_^+^ drives this increase in MPB productivity as NH_4_^+^ uptake was higher in +AS plots at the sandy site, especially in summer. There was also a trend for greater denitrification potential in +AS sandy site plots in summer. At the muddy-sand site there was no significant effect of *Austrovenus* on GPP, NH_4_^+^ uptake or denitrification potential. Furthermore, GPP and denitrification potential were both significantly lower than at the sandy site.

As for other suspension feeding bivalves in coastal systems worldwide, resuspended MPB are an important component of *Austrovenus*' diet, especially as water column primary productivity is typically low in New Zealand estuaries [46], [47], [48]. Previous research with *Austrovenus* and other large bioturbating macrofauna has also observed an increase in MPB productivity even though MPB are often a major food source for the animals [12], [14], [25]. However, this study suggests that the positive effect of *Austrovenus* on MPB productivity is not consistent across habitat types, and that there can be substantial temporal variability in GPP. At both sites, the lower rates of GPP in winter are likely to be caused by limited MPB and bivalve activity. MPB photosynthetic activity was likely limited by wintertime water temperatures and reduced levels of PAR, while the reduced dark chamber NH_4_^+^ fluxes in the wintertime *Austrovenus* addition treatments provided evidence of reduced metabolic rates (i.e. less NH_4_^+^ excretion during the colder winter period). More surprising are the low rates of GPP in summer, and lack of an effect of *Austrovenus* on GPP, at the muddy-sand site. As dark chamber NH_4_^+^ fluxes in +AS plots were similar between the two sites it seems unlikely that the reason for low MPB productivity at the muddy-sand site was nutrient limitation.

Muddy sediments, despite often having higher microalgal biomass, can be less productive (in terms of rates of photosynthesis and oxygen evolution) than sandy sediments [49]. Resuspension of fine sediments, causing light limitation at the benthos, is more likely in muddy sediments, but we did not observe higher levels of turbidity at our muddier site on the days that we sampled. However, productivity can be enhanced in sandy sediments because light can penetrate further into the sediment column (as there is greater interstitial space between sediment grains). This increased sediment permeability can enhance solute flux (by permitting pore-water advection), and more frequent resuspension can cause a higher turnover of algal biomass [49], [50], [51]. Furthermore, sediment grain size can affect microbial composition and activity, and thus organic matter remineralisation, nutrient availability and MPB productivity [51]. In fact, we found normalised GPP was significantly increased at the sandy site compared to the muddy-sand site even in -AS plots, although the effect was enhanced in +AS plots.

At the sandy site other macrofaunal abundance and biomass in +AS plots was dominated by mudflat anemones (*Anthopleura aureoradiata*). Previous work has described a mutualistic relationship between *Austrovenus* and *A. aureoradiata* whereby the anemones use the living bivalves as hard substrate for attachment and the bivalves gain protection from parasitic infection [52]. The anemones may also benefit from greater NH_4_^+^ availability in *Austrovenus* beds as endosymbiotic zooxanthellae can uptake NH_4_^+^ from surrounding water [53]. It is probable that mudflat anemones significantly contribute to, and complicate, nutrient recycling and productivity at the sandy site, by both excretion and uptake of NH_4_^+^, but further work is needed to determine whether this species is a net source or sink of NH_4_^+^, and its effect on system productivity. Barnacles were also supported on *Austrovenus* shells at the sandy site. It is therefore possible that the positive effect on productivity measured in +AS plots at the sandy site is not attributable to *Austrovenus* alone, but to the combination of *Austrovenus* and the macrofaunal communities they support.

In contrast, at the muddy-sand site other macrofaunal abundance and biomass was dominated by other bivalves (mostly the deposit feeders *Nucula hartvigiana* and *Macomona liliana*). We expected OM content to increase in +AS plots at this site, due to retention of biodeposits in the lower energy environment, but instead found the reverse to be true. Deposit-feeder abundance and biomass was higher in +AS plots and they may have utilised the increased supply of OM. Alternatively, decreased mud content and increased grain size in +AS plots suggests that *Austrovenus* bioturbation enhanced fine sediment and OM transport by destabilising the sediment [54], [55]. Furthermore, there was no difference in sediment C, N and C:N ratio between +AS and -AS plots. Typically, these parameters are found to increase under epifaunal bivalve beds, particularly so under longline mussel farms [34], [56], [57]. Although biodeposition rates would almost certainly be lower for an infaunal bivalve bed than for a three dimensional epifaunal bed/longline our results suggest that *Austrovenus* biodeposits do not accumulate at either site. It is probable that OM is dispersed by currents at the sandy site, and quickly utilised by deposit feeders and/or resuspended by bivalve bioturbation at the muddy-sand site.

Unamended sediment denitrification rates were nil or low, likely because sediment nitrification may be a major source of NO_3_^-^ for denitrification and coupled nitrification-denitrification is inhibited by our method (e.g. [58]). This is further reinforced by the low measured NO_3_^-^ fluxes into the sediment. Our expectation was that increased N from *Austrovenus* biodeposits (at the more sheltered muddy-sand site especially) would fuel coupled nitrification-denitrification but we found that OM content was not increased in +AS plots, and denitrification remained N limited regardless of site, season or addition/removal of *Austrovenus.* However, sediment denitrification potential (as measured with excess nitrate and carbon) did trend towards an increase in +AS plots at the sandy site in summer. Although *Austrovenus* biodeposits may not accumulate at the sandy site, bivalve bioturbation and excretion may have enhanced NH_4_^+^ availability, thus providing a source of nitrogen for nitrification [59], [60]. NH_4_^+^ uptake was significantly increased in +AS plots at the sandy site in summer, which may have been partly due to increased nitrification. Without measuring sediment nitrification rates, however, it is not possible to separate uptake by nitrifiers from that by MPB (and perhaps anemones also). Nitrifiers are known to be poor competitors for nitrogen [61], [62], but oxygen production by benthic photosynthesis may enhance rates of coupled nitrification-denitrification when NH_4_^+^ is not limiting [63]. Our results suggest that increased availability of NH_4_^+^ at the sandy site in summer as a result of *Austrovenus* activity likely increases both MPB productivity and sediment denitrification, though concurrent measurements of GPP, nitrification and denitrification would be needed to confirm this.

A possible confounding factor influencing the interpretation of our results is the difference in *Austrovenus* size between the two sites. Individuals were significantly larger at the sandy site (c. 23 mm shell length) than at the muddy-sand site (c. 18 mm shell length). Previous research has indicated that *Austrovenus* condition is enhanced in sandy compared to muddier sediments [64], and the bivalves in our experimental plots had been transplanted from nearby beds at each site so represented a natural size for the habitat type. As biomass was comparable between our sites we would not expect first order excretion rates to be substantially different between sites. However, the size difference might affect the degree to which *Austrovenus* bioturbation alters sediment chemistry. Bioturbation by macrofauna that mix surficial sediments, such as *Austrovenus*, can facilitate the release of solutes from sediment porewater [45]. Previous experiments have shown that *Austrovenus* tend to be retained in unfenced high-density plots, i.e. individual bivalves display minimal horizontal movement through surface sediments when in high-density beds [23], [25]. The main effect of *Austrovenus* bioturbation in bivalve beds is therefore likely to be small-scale (< 2–3 cm) vertical movement as the bivalves move to the sediment-water interface to feed around high tide, and thereafter retreat to just below the sediment surface. The larger bivalves at our sandy site may have reworked sediment to a greater depth than the smaller individuals at our muddy-sand site. However, solute gradients are likely to be steeper in sediments at the muddy-sand site, potentially offsetting the size difference, and making it difficult to speculate on size-specific bioturbation effects on solute fluxes.

It is well documented that denitrification is often highly variable over small spatial and temporal scales in estuaries, due to variable O_2_ profiles, nitrate and OM availability in the sediment [40], [65]. This is caused by a variety of processes such as frequent wetting/drying due to the tides or macrofauna activity (especially bioturbation and burrow building) which create anoxic denitrification microsites and make collection of a large number of replicates crucial [65]. More sophisticated (but more expensive) techniques, such as isotope-pairing techniques using Membrane Inlet Mass Spectrometry, can quantify denitrification rates without blocking nitrification, which may help to resolve the complicated interactions among macrofauna, such as *Austrovenus*, MPB and microbial communities (e.g. [66], [67]). Our work shows that these interactions are likely to be further complicated by context (i.e. spatial and temporal variability), so future studies should be mindful of this.

There is typically a trade-off between the size of experimental plots and the number of replicates that can be established. We recognise the low levels of replication (n = 3 per treatment) inherent in our experiments, but our efforts were focused on using relatively large plots as the estuarine intertidal is dynamic and subject to substantial bedload transport and sediment reworking rates (e.g. [68]); consequently results from experiments using smaller-scale manipulations may be dominated by edge effects [69], [70]. Furthermore, modifications of sediment stability associated with the addition or removal of macrofauna are often scale and/or density dependent [71], [72], [73]. We recognise also that there are limitations associated with using benthic chambers to measure solute fluxes, such as stirring-induced pressure gradients that affect rates of porewater exchange [74], or altered boundary layer dynamics [75]. However, in sediments colonised by large bioturbating or bioirrigating macrofauna and by patchy MPB communities (as in this study) there is considerable small-scale spatial and temporal heterogeneity in solute distribution. Benthic chambers have the advantage of integrating fluxes over a large sediment surface area, and in this study our intention was to identify any differences in relative fluxes between our sites, seasons and treatments, rather than quantifying absolute fluxes.

### Conclusions

*Austrovenus* enhanced primary productivity and sediment denitrification potential at the sandy site, whereas there was no effect of *Austrovenus* on these variables at the muddy-sand site, leading us to hypothesise that increasing estuarine mud content may limit the influence of this key species on ecosystem function. However, there is a need to sample across a gradient of increasing muddiness to further explore these relationships. Similarly, there is a need for more comprehensive sampling to better resolve temporal variability. Previous research has established that high levels of sedimentation are likely to reduce *Austrovenus* populations [8], but our results indicate that moderate levels of sedimentation may reduce the positive effect of this species on system productivity even when they persist. Furthermore, our results suggest that denitrification potential is lower in muddy-sand compared to sandy sediments so moderate levels of sedimentation may also limit the system's ability to counteract the effects of eutrophication. The study reveals that it is important to consider context, i.e. the range of conditions inhabited by a particular species, in order to assess the effect of key species on ecosystem function. It appears that it is not just the loss of key species, but alteration of those species' habitats (even without substantial changes in biomass), that has the potential to alter ecosystem function.
